# Etoposide/platinum plus anlotinib for patients with transformed small-cell lung cancer from EGFR-mutant lung adenocarcinoma after EGFR-TKI resistance: a retrospective and observational study

**DOI:** 10.3389/fonc.2023.1153131

**Published:** 2023-06-09

**Authors:** Jianghua Ding, Zhaohui Leng, Hong Gu, Xiang Jing, Yun Song

**Affiliations:** ^1^ Department of Hematology & Oncology, Jiujiang University Affiliated Hospital, Jiujiang, Jiangxi, China; ^2^ Department of Hematology & Oncology, Ruichang People Hospital, Ruichang, Jiangxi, China; ^3^ Department of Hematology & Oncology, Lushan People Hospital, Lushan, Jiangxi, China

**Keywords:** etoposide/platinum (EP), anlotinib, transformation, small-cell lung cancer (SCLC), lung adenocarcinoma (LUAD), epidermal growth factor receptor (EGFR)

## Abstract

**Objective:**

The histological conversion of lung adenocarcinoma (LUAD) into small-cell lung cancer (SCLC) is an important resistance mechanism for epidermal growth factor receptor (EGFR)-tyrosine kinase inhibitor (TKI)-resistant LUAD. Anlotinib has been recommended as the third-line treatment for SCLC patients. The efficacy of etoposide/platinum (EP) as the main treatment is very limited for patients with transformed SCLC. However, little is known about EP plus anlotinib for transformed SCLC. The present study retrospectively explored the clinical response to EP combined with anlotinib in patients with transformed SCLC from LUAD after EGFR-TKI failure.

**Methods:**

A total of 10 patients who underwent SCLC transformation from EGFR-TKI-resistant LUAD were retrospectively reviewed from September 1, 2019, to December 31, 2022, in three regional hospitals. All of the patients were treated with the combination regimen of EP and anlotinib for four to six cycles, followed by anlotinib maintenance therapy. The clinical efficacy indices including objective response rate (ORR), disease control rate (DCR), median progression-free survival (mPFS), median overall survival (mOS), and toxicities were evaluated.

**Results:**

The median time from EGFR-TKI treatment to SCLC conversion was 20.1 ± 2.76 months (17–24 months). Genetic examination after transformation showed that 90% of the patients retained their original EGFR gene mutations. Additional driver genes were found, including BRAF mutation (10%), PIK3CA mutation (20%), RB1 loss (50%), and TP53 mutation (60%). The ORR and DCR were 80% and 100%, respectively. The mPFS was 9.0 months (95% CI, 7.9–10.1 months), and the mOS was 14.0 months (95% CI, 12.0–15.9 months). Less than 10% of grade 3 toxicities were observed, and no grade 4 toxicity and death events were reported.

**Conclusion:**

The EP plus anlotinib regimen appears to be a promising and safe strategy in transformed SCLC patients after EGFR-TKI resistance, which warrants further investigation.

## 1 Introduction

Epidermal growth factor receptor (EGFR) is the most prominent driving gene in non-small-cell lung cancer (NSCLC), mainly including EGFR exon 19 deletion and L858R mutation. EGFR-tyrosine kinase inhibitors (EGFR-TKIs) have been listed as the preferable standard of care in EGFR-mutant NSCLC patients, in particular for lung adenocarcinoma (LUAD). However, nearly all patients inevitably experience acquired resistance to EGFR-TKI. Among these patients, 5–15% display histological transformation from NSCLC to small-cell lung cancer (SCLC) (1). The underlying mechanisms are very complicated, and most of them remain unclear. For transformed SCLC, chemotherapy with etoposide/platinum (EP) is the most common regimen, but the clinical prognosis is dismal, with a median overall survival (mOS) of merely 6–10.9 months (2, 3).

As the principal angiogenic growth factor, vascular endothelial growth factor (VEGF) modulates the process of angiogenesis during the growth, invasion, and metastasis of tumors (4). In SCLC, antiangiogenic agents targeting VEGF have not become an important therapeutic strategy until the advent of anlotinib. As an oral antiangiogenic tyrosine kinase inhibitor (TKI), anlotinib has been recommended by Chinese Society of Clinical Oncology (CSCO) as a third-line treatment for SCLC and NSCLC (5, 6). Majority of patients with transformed SCLC after EGFR-TKI resistance of LUAD have received more than one systemic treatment. Furthermore, the combination of anlotinib with EP regimen has been administered as the first-line treatment of extensive-stage SCLC with an objective response rate (ORR) of 87.2% and a median progression-free survival (mPFS) of 9.0 months (7). However, little is known about the efficacy of the combination regimens in transformed SCLC from EGFR-TKI-resistant LUAD. Therefore, this study retrospectively analyzed the clinical efficacy and safety of the EP regimen plus anlotinib for patients with the histological conversion from EGFR-TKI-resistant LUAD to SCLC.

## 2 Materials and methods

### 2.1 Study design and patients

This was a multicenter retrospective observational study. All transformed SCLC patients who received anlotinib combined with EP chemotherapy were from three regional hospitals, namely the Affiliated Hospital of Jiujiang University, Ruichang People Hospital, and Lushan People Hospital, between September 1, 2019, and December 31, 2022. The clinical data of the patients were collected, including age, sex, ECOG PS, histopathology, molecular examination, TNM stage, anlotinib dose, and adverse reaction.

### 2.2 Inclusion and exclusion criteria

The inclusion criteria for patients were as follows: (1) 18–75 years of age; (2) pathologically confirmed histological transformation from EGFR-mutant LUAD to SCLC; (3) more than one systemic treatment of EGFR-TKI before transformation; (4) ECOG PS ≤ 2; and (5) TNM stage: IIIB–IV; (6) no obvious abnormality in liver and kidney function; (7) no obvious hematological abnormality; (7) no active bleeding and coagulation abnormalities; (8) no clinically significant electrocardiograph abnormality.

The exclusion criteria for patients were as follows: (1) age >75 years; (2) initial histopathological diagnosis of SCLC; (3) ECOG PS >2; and (4) presentation of active bleeding; (5) presence of contraindications to chemotherapy.

### 2.3 Next-generation sequencing

DNA was extracted from tumor tissue and matched pleural fluid samples. Next-generation sequencing (NGS) was performed *via* a panel of at least 73 genes in Daan Gene Co., Ltd. (Guangzhou, China), covering all exons of EGFR gene with a mean coverage depth of >800×.

### 2.4 Therapeutic methods

All of the patients were treated with a combination regimen of EP and anlotinib, i.e., 80 mg/m^2^ etoposide (Qilu Phar., Jinan, China) for days 1–3, carboplatin (Qilu Phar., Jinan, China) (AUC = 5) on day 1, and anlotinib (10 mg/day) (Chia-Tai Tianqing Phar., Nanjing, China) orally for days 1–14. The cycle was repeated every 3 weeks for four to six cycles, and then anlotinib was maintained every 21 days. Dose adjustment was made according to the patients’ actual situation. The treatment was terminated if disease progression, death, or unacceptable toxicity occurred.

### 2.5 Efficacy and safety evaluation

The clinical efficacy was evaluated according to the RECIST standard (ver. 1.1). The objective responses were classified as complete remission (CR), partial remission (PR), stable disease (SD), and progressive disease (PD). The primary end points were ORR (CR + PR) and mPFS, and the secondary end points were DCR (CR + PR + SD) and median overall survival (mOS).

The adverse reaction grades were classified following the Common Terminology Criteria for Adverse Events (CTCAE) (ver. 5.0).

### 2.6 Follow-up and statistical analysis

PFS was defined as the period from the initial date of chemotherapy plus anlotinib to disease progression or death. OS was determined from the start of chemotherapy with anlotinib to death or the date of last follow-up evaluation. Time to SCLC transformation was calculated from the initial date of EGFR-TKI treatment to confirmation of transformed SCLC.

The cutoff date for follow-up was December 31, 2022. The Kaplan–Meier method was used to analyze the median PFS, OS, and 95% confidence interval (CI). All of the statistical analyses were performed using Statistical Package for the Social Sciences (SPSS, ver. 20.0, Chicago, Illinois, U.S.A) and GraphPad Prism (ver. 7.0, San Diego, California, U.S.A).

## 3 Results

### 3.1 Baseline clinical features of patients

Out of 152 patients with EGFR mutations, a total of 10 patients (6.57%) with transformed SCLC were enrolled in the present study. Their baseline clinical features are given in **Tables 1**, **2**. All of the included patients were in IIIB–IVB stage. The initial mutation status included EGFR exon 19 Del (60%, 6/10) and EGFR exon 21 L858R mutation (40%, 4/10). One patient (no. 10) had a concurrent T790M mutation. 80% (8/10) of the patients received osimertinib (AstraZeneca Phar., London, UK) as the first-line treatment, while only 20% (2/10) of the patients received aumolertinib (HanSoh Phar., Lianyungang, China) treatment. The median interval from initial treatment to transformation was 20.1 ± 2.76 months (17–24 months).

**Table 1:** Baseline clinical features of 10 patients with transformed SCLC from EGFR-mutant LUAD.

Case No.	Age (Years)	Smoking status	Gender	Stage	Initial mutation status (specimen type)	Primary tumor lesion	TKI therapy before transformation	Time to SCLC transformation (months)	Comorbidities	Specimen type (2^nd^ NGS)
1	62	Yes	Male	IIIB	EGFR exon 19 del (tissue)	left lower lung	Osimertinib	19	chronic bronchitis	tissue
2	54	Never	Female	IVA	EGFR exon 19 del (tissue)	right lower lung	Osimertinib	22	none	tissue
3	46	Never	Male	IVB	EGFR exon 21 L858R, (tissue)	right middle lung	Osimertinib	17	none	pleural fluid
4	59	Yes	Male	IIIB	EGFR exon 19 del, (tissue)	left upper lung	Aumolertinib	23.5	chronic bronchitis	lymph node
5	67	Yes	Female	IIIB	EGFR exon 19 del, T790M (+) (tissue)	right lower lung	Aumolertinib	21	obstructive emphysema	tissue
6	38	Yes	Male	IVB	EGFR exon 21 L858R, (tissue)	right lower lung	Osimertinib	16	chronic bronchitis	pleural fluid
7	46	Yes	Male	IIIB	EGFR exon 19 del, (tissue)	left lower lung	Osimertinib	22.5	chronic bronchial asthma	tissue
8	61	Yes	Female	IVA	EGFR exon 21 L858R, (tissue)	right upper lung	Osimertinib	19.5	chronic bronchial asthma	lymph node
9	57	Never	Male	IVA	EGFR exon 19, (tissue)	right lower lung	Aumolertinib	20.5	diabetes mellitus	lymph node
10	59	Never	Male	IVB	EGFR exon 21 L858R, T790M (+) (tissue)	left lower lung	Osimertinib	25.5	hypertension	tissue

**Table 2:** The post-transformation gene mutations of NGS in the 10 patients.

Gene (Mutation point)	Patients (Mutation abundance (%))									
	Patient 1	Patient 2	Patient 3	Patient 4	Patient 5	Patient 6	Patient 7	Patient 8	Patient 9	Patient 10
EGFR	Exon 19 del	Exon 19 del	Exon 21 L858R	✘	Exon 19 del	Exon 21 L858R	Exon 19 del	Exon 21 L858R	Exon 19 del	Exon 21 L858R
T790M	✘	✘	✘	✘	✓(12.4%)	✘	✘	✘	✘	✓(17.6%)
RB1 loss	✓(9.2%)	✘	✓(6.5%)	✓(13.4%)	✓(15.2%)	✘	✓(5.7%)	✓(7.4%)	✘	✓(13.7%)
TP53	✓ (p.A159D) (8.4%)	✓(p.R273H)(13.7%)	✓(p.R273H) (20.3%)	✓(p.A159D) (17.8%)	✓(p.A159D) (5.9%)	✘	✓(p.A159D) (7.6%)	✓(p.R273H) (13.4%)	✓(p.R273H) (22.8%)	✓(p.A159D) (14.6%)
MYC amplification	✘	✘	✘	✘	✘	✘	✘	✘	✓(28.4%)	✘
NF1 (p.R461T)	✘	✘	✘	✘	✘	✓(22.5%)	✘	✘	✘	✘
PIK3CA (p.E545K)	✘	✘	✘	✘	✘	✘	✓(4.6%)	✘	✘	✘
PTEN loss	✓(16.8%)	✘	✘	✘	✘	✘	✘	✘	✘	✘
CCNE1 (Exon7, c.476A > G)	✘	✘	✘	✓(8.9%)	✘	✘	✘	✘	✘	✘
CDK6 amplification	✘	✓(14.2%)	✘	✘	✘	✘	✘	✘	✘	✘
BRAF (p.D594G)	✘	✘	✓ (5.6%)	✘	✘	✘	✘	✘	✘	✘

✓ indicating the presence of gene mutation, ✘ indicating the absence of gene mutation.

All of the patients underwent the second genetic testing, and the specimens included tissue, pleural fluid, and lymph node. Compared with the initial gene mutation, the second mutation status of transformed SCLC was very complicated. Except for patient no. 4, who lost the initial EGFR exon 19 deletion, the nine remaining patients retained their original EGFR gene mutations. These mutations were accompanied by additional driver gene mutations, including TP53 mutation (60%), RB1 loss (50%), PIK3CA mutation (20%), BRAF mutation (10%), PTEN (10%), CDK6 (10%), CCNE(10%), NF1 (10%) and MYC (10%). Of note, patient no. 3 carried TP53 mutation but did not experience RB1 loss, while patient no. 10 harbored RB1 loss and TP53 mutation but lost T790M mutation.

### 3.2 Clinical efficacy

All the patients discontinued osimertinib or aumolertinib treatment after disease progression, and then receive the combination treatment of EC plus anlotinib. In the present study, four patients were in stage IIIB, but they exhibited poor performance status (PS=2) due to the comorbidities including as chronic bronchitis (no. 1 and 4), obstructive emphysema (no.5), and chronic bronchial asthma (no.7), respectively. So, they only received EGFR-TKI therapy alone without thoracic radiotherapy.

**Figure 1:**
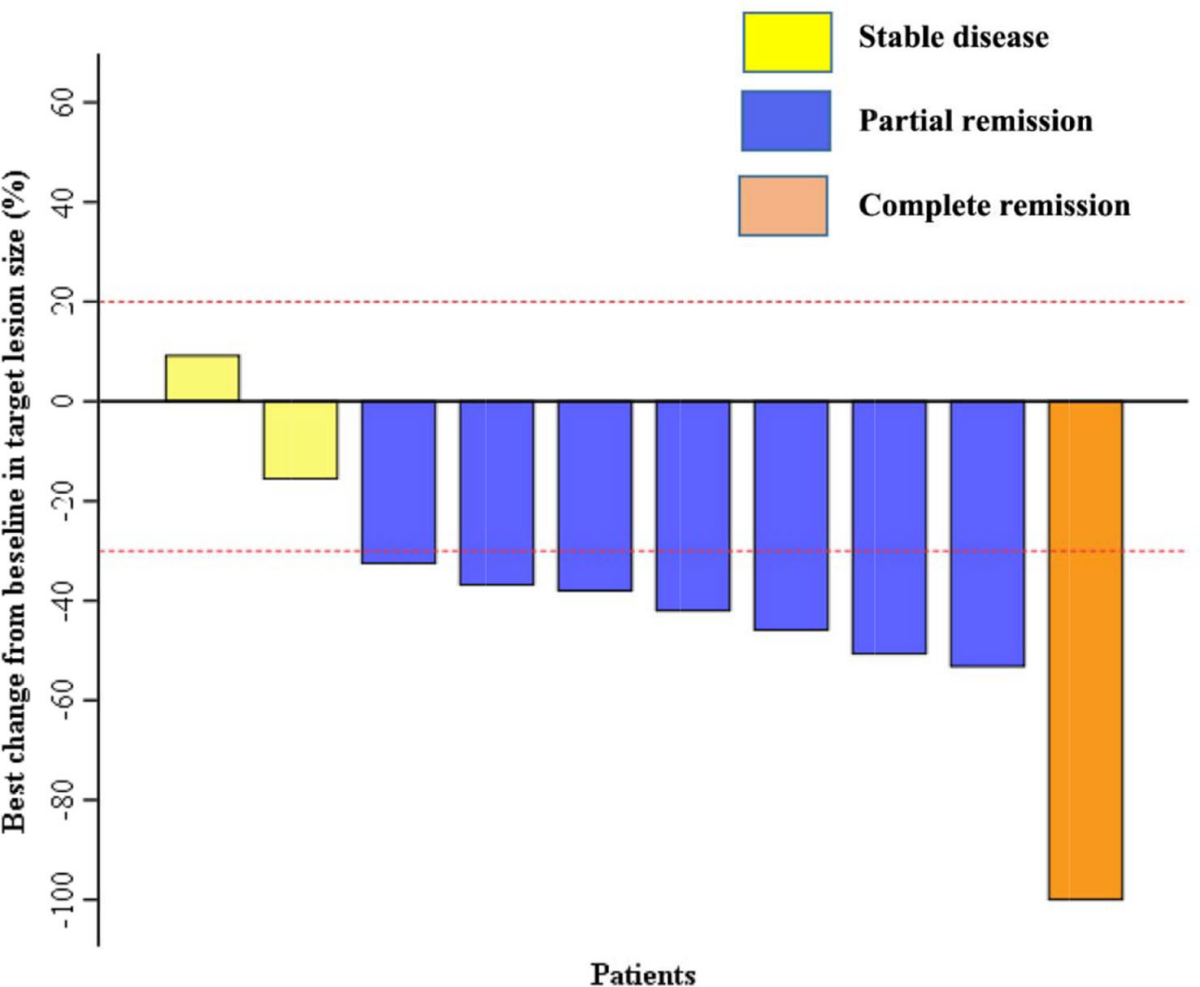
Changes from baseline in target lesions size (%).

**Figure 2:**
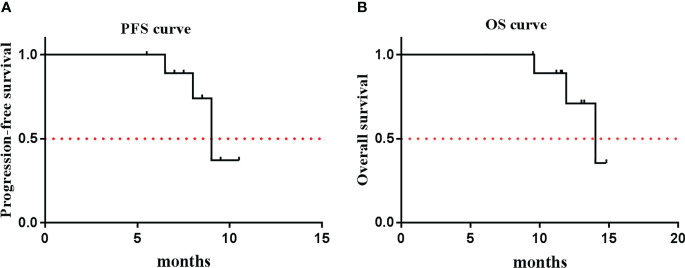
Kaplan-Meier Estiamtes of survival. **(A)** PFS: 9.0 months (95% CI: 7.9~10.1); **(B)** OS: 14.0 months (95% CI: 12.0~15.9).

Except for one patient (no. 8), who only received four cycles of the combination treatment, the remaining nine patients received six cycles of EP and anlotinib treatment. All of the patients received anlotinib as maintenance therapy after the completion of the combination treatment. One patient achieved CR, seven patients achieved PR, and two patients had SD (**Figure 1**). The ORR was 80%, and the DCR was 100% (**Table 3**). The median PFS was 9.0 months (95% CI, 7.9–10.1 months), and the median OS was 14.0 months (95% CI, 12.0–15.9 months) (**Figure 2**). The median follow-up time was 15.2 months (95% CI, 13.4-16.8 months).

**Table 3:** Clinical outcome of transformed SCLC from LUAD with EP plus anlotinib.

Clinical efficacy	Number (%)
CR	1 (10%)
PR	7 (70%)
SD	2 (20%)
PD	0 (0%)
ORR (CR+PR)	8 (80%)
DCR (CR+PR+SD)	10 (100%)

CR, complete remission; PR, partial remission; SD, stable disease; PD, progressive disease; ORR, overall response rate; and DCR, disease control rate.

### 3.3 Safety

All of the patients were included in the safety assessment. Adverse reactions were assessed from the start of the combination treatment until disease progression or the last follow-up date. The treatment-related adverse effects included vomiting and nausea, granulocytopenia, leukopenia, thrombocytopenia, hypertension, proteinuria, fatigue, hand–foot syndrome, and leukopenia (**Table 4**). The grade 3 toxicities were granulocytopenia (10%), leukopenia (5%), and hypertension (10%). No grade 4 toxicities were recorded, and no deaths were observed.

**Table 4:** Treatment-related adverse effects (n (%)).

Adverse effects	No. of patients				Total
	Grade 1	Grade 2	Grade 3	Grade 4	
Vomiting and nausea	3 (30%)	1 (10%)	0	0	4 (40%)
Granulocytopenia	4 (40%)	1 (10%)	1 (10%)	0	6 (60%)
Leukopenia	5 (50%)	2 (20%)	1 (10%)	0	8 (80%)
Thrombocytopenia	4 (40%)	1 (10%)	0	0	5 (50%)
Hypertension	6 (60%)	1 (10%)	1 (10%)	0	8 (80%)
Proteinuria	2 (20%)	1 (10%)	0	0	3 (30%)
Fatigue	5 (50%)	2 (20%)	0	0	7 (70%)
Oral mucositis	4 (40%)	1 (10%)	0	0	5 (50%)
Hand-foot syndrome	3 (30%)	1 (10%)	0	0	4 (40%)

## 4 Discussion

In 2006, a female NSCLC patient carrying EGFR exon 19 deletion was first reported to transform to SCLC (8). Since then, cases of LUAD conversion to SCLC have been continually presented (9–11). Statistically, 4–14% of EGFR-mutant NSCLC patients experience histological transformation to SCLC after EGFR-TKI failure. The histological conversion to SCLC represents one of the important mechanisms governing EGFR-TKI resistance. Three prevailing mechanisms may be proposed to explain the histological conversion from LUAD to SCLC. First, twin clones (i.e., both LUAD and SCLC clones) coexist in the tumor sites in the initial stages of tumorigenesis. LUAD cells are the dominant clones during the early stage, which become constrained under the pressure of EGFR-TKI treatment. Accordingly, the new clones of SCLC emerge and replace the previously predominant clones. Second, both SCLC and LUAD originate from the common precursor, i.e., alveolar type II cells. With long exposure to EGFR-TKI, the resistant clones survive and then convert to SCLC type. Finally, secondary gene alterations appear in the process of transformation, including RB1 loss, TP53 mutation, and PIK3CA and BRAF mutation. Recent studies have revealed some novel gene alterations that are associated with the course of transformation, which include WNK1 mutation (12), SPP1 upregulation (13), REST inactivation (14), and ETV1 mutation (15). In addition, Xie et al. reported that the conversion of LUAD to SCLC may result from somatic copy number variation (CNV) events rather than from mutational events. The burden of CNV is closely associated with the interval time to transformed SCLC and OS after SCLC conversion (16). The definitive mechanisms behind the histological transformation are very complicated and remain to be fully clarified.

Currently, an increasing number of researchers prefer the shared-origin theory. Logistically, if the theory of twin clones is true, it is difficult to explain PR or even CR response to first-line EGFR-TKI treatment. In the present study, 80% of the patients achieved more than PR response (including CR in one patient). Importantly, 90% of the patients retained their prior EGFR gene mutations. These results strongly support the common-precursor doctrine of LUAD and SCLC. Furthermore, RB1 loss was found in 50% of the patients, and TP53 mutation occurred in 60% of the patients. One patient (no. 3) harbored only gene alterations of TP53 but without RB1 loss. These findings indicate that RB1 loss and TP53 mutation are not universally present in transformed SCLC patients, which has also been confirmed by others (12, 17). Additionally, PIK3CA mutation was found in 20% of the patients, and BRAF mutation occurred in 10% of the patients, suggesting the molecular heterogeneity of transformation from LUAD to SCLC. Finally, the interval time from EGFR-TKI treatment to histological transformation was 20.1 months, which is consistent with previous reports of 17.8–22.7 months (2, 12, 18).

Currently, antiangiogenesis therapy targeting VEGF has become an indispensable strategy for cancer treatment. VEGF overexpression has been found in almost 80% of SCLC patients, indicating highly vascularized tumor of SCLC. The anti-VEGF agents, such as thalidomide, sorafenib, and sunitinib, showed disappointing clinical efficacy but increased treatment-related toxicity (19–21). In extensive-stage SCLC patients, bevacizumab plus EP regimen prolonged the PFS (6.7 vs. 5.7 months, P=0.03) but didn’t translate into the benefit of OS (8.9 vs. 9.8 months, P=0.113) compared with EP regimen (22). Obviously, the role of antiangiogenic drugs remains controversial in the treatment of SCLC until the advent of anlotinib.

In ALTER 1202 study, the novel antiangiogenic agent anlotinib as a third- or further-line treatment achieved better mPFS (4.1 vs. 0.7 months, P < 0.0001) and mOS (7.3 vs. 4.9 months, P = 0.0029) than the placebo group for patients with extensive-stage SCLC (ES-SCLC) (23). Consequently, the Chinese Society of Clinical Oncology (CSCO) recommended anlotinib as the only antiangiogenic agent for refractory ES-SCLC in China on August 30, 2019. Furthermore, a prospective study of ACTION-2 reported that EP plus anlotinib regimen as the first-line treatment for ES-SCLC achieved an ORR of 87.2%, a DCR of 97.7%, an mPFS of 9.0 months, and an mOS of 19.0 months (7). A single-arm trial showed that anlotinib plus EP as the first-line treatment for ES-SCLC achieved an ORR of 85.71%, a DCR of 94.29%, an mPFS of 8.02 months, and an mOS of 15.87 months (24). Inspired by this, we attempted to explore the combination of EP with an anlotinib regimen in transformed SCLC patients.

In *de novo* extensive SCLC, immune-combination therapy has been recommended as the first-line treatment with mOS reaching 13–15.4 months (25–27). Conversely, no objective responses were observed in 17 transformed SCLC cases who received immunotherapy (2). The EP regimen is the most common therapy for the transformed SCLC but with limited efficacy (only 3.2–4.0 months of mPFS and 8.0–10.9 months of mOS) (2, 12, 18, 28, 29). Wang et al. reported that the ORR and mPFS of EP chemotherapy for transformed SCLC were 44.4% and 3.5 months, respectively, but the ORR and mPFS of anlotinib alone were 66.7% and 6.2 months, respectively, indicating anlotinib as an optional choice in this population (29). In the present study, the combination of EP with an anlotinib regimen was used to treat transformed SCLC patients. The mPFS and mOS were 9.0 months (95% CI, 7.9–10.1 months) and 14.0 months (95% CI, 12.0–15.9 months), respectively. Additionally, the combination regimen was associated with a favorable safety profile, with less than 10% of grade 3 toxicities and no grade 4 toxicities and deaths. Therefore, EP plus anlotinib regimen in our study seems to achieve higher clinical efficacy than EP chemotherapy or anlotinib alone in previous studies.

## 5 Conclusions

Heretofore, no treatment guidelines have been established for the transformed SCLC. Our study revealed that EP combined with anlotinib may be a better choice for transformed SCLC originating from EGFR-TKI-resistant LUAD compared with EP or anlotinib alone treatment. However, this study also has some shortcomings. On the one hand, the sample size was small. On the other hand, the present study was retrospective in nature, and the results were observational. Due to the study limitations, further well-designed prospective studies with large sample sizes should be performed to confirm these findings.

## Data availability statement

The raw data supporting the conclusions of this article will be made available by the authors, without undue reservation.

## Ethics statement

The studies involving human participants were reviewed and approved by Ethics Committee of Jiujiang University Affiliated Hospital. The patients/participants provided their written informed consent to participate in this study. Written informed consent was obtained from the individual(s) for the publication of any potentially identifiable images or data included in this article.

## Author contributions

Conceptualization and writing: JD. Patients’ data collection: ZL, HG and XJ. Data processing: YS. All of the authors have read and agreed to the published version of the manuscript. All authors contributed to the article and approved the submitted version.
